# Synergistic effect of Indian hedgehog and bone morphogenetic protein-2 gene transfer to increase the osteogenic potential of human mesenchymal stem cells

**DOI:** 10.1186/scrt316

**Published:** 2013-09-02

**Authors:** Johannes C Reichert, Jonas Schmalzl, Patrick Prager, Fabian Gilbert, Verena MC Quent, Andre F Steinert, Maximilian Rudert, Ulrich Nöth

**Affiliations:** 1 Department of Orthopaedic Surgery, König-Ludwig-Haus, Center for Musculoskeletal Research, Julius-Maximilians-University, Brettreichstraße 11, 97074 Würzburg, Germany; 2 Department of Obstetrics and Gynecology, University Hospital Erlangen, Friedrich-Alexander University Erlangen-Nuremberg, Erlangen, Germany

## Abstract

**Introduction:**

To stimulate healing of large bone defects research has concentrated on the application of mesenchymal stem cells (MSCs).

**Methods:**

In the present study, we induced the overexpression of the growth factors bone morphogenetic protein 2 (BMP-2) and/or Indian hedgehog (IHH) in human MSCs by adenoviral transduction to increase their osteogenic potential. GFP and nontransduced MSCs served as controls. The influence of the respective genetic modification on cell metabolic activity, proliferation, alkaline phosphatase (ALP) activity, mineralization in cell culture, and osteogenic marker gene expression was investigated.

**Results:**

Transduction had no negative influence on cell metabolic activity or proliferation. ALP activity showed a typical rise-and-fall pattern with a maximal activity at day 14 and 21 after osteogenic induction. Enzyme activity was significantly higher in groups cultured with osteogenic media. The overexpression of BMP-2 and especially IHH + BMP-2 resulted in a significantly higher mineralization after 28 days. This was in line with obtained quantitative reverse transcriptase polymerase chain reaction (qRT-PCR) analyses, which showed a significant increase in *osteopontin* and *osteocalcin* expression for osteogenically induced BMP-2 and IHH + BMP-2 transduced cells when compared with the other groups. Moreover, an increase in *runx2* expression was observed in all osteogenic groups toward day 21. It was again more pronounced for BMP-2 and IHH + BMP-2 transduced cells cultured in osteogenic media.

**Conclusions:**

In summary, viral transduction did not negatively influence cell metabolic activity and proliferation. The overexpression of BMP-2 in combination with or without IHH resulted in an increased deposition of mineralized extracellular matrix, and expression of osteogenic marker genes. Viral transduction therefore represents a promising means to increase the osteogenic potential of MSCs and the combination of different transgenes may result in synergistic effects.

## Introduction

The majority of bone defects and fractures show spontaneous healing, stimulated by well-orchestrated endogenous cell populations and microenvironmental cues. Generally, treatment outcomes have improved because of advances regarding surgical techniques and implant design. However, different factors, such as biomechanical instability and extensive soft tissue trauma, can ultimately promote the formation of nonunions and defects characterized by a restricted regenerative potential [1]. Such bone defects are associated with considerable surgical challenges and have a high socioeconomic impact, greatly curtailing affected patients’ quality of life [2,3]. The augmentation of large bone defects usually requires the application of autologous bone graft (ABG). Limiting associated factors include graft availability, comorbidity, and insufficient bony integration.

Research in the field has therefore concentrated on the application of mesenchymal stem cells (MSCs), which are able to differentiate into bone-forming osteoblasts [4]. Successful translation of tissue engineering concepts to the field of oral and maxillofacial surgery has suggested the extension to long bone regeneration [5,6]. However, promising results obtained in vitro or in small animal models could seldom be reproduced in the human organism [7].

In the present study, we therefore induced the overexpression of the growth factors bone morphogenetic protein 2 (BMP-2) and/or Indian hedgehog (IHH) in human MSCs by adenoviral transduction to increase their osteogenic potential.

The beneficial effects of BMP-2 on osteogenic differentiation are well documented [8]. The hedgehog pathway plays a critical role in skeletogenesis, controlling self-renewal, migration, differentiation, and cell-fate commitment of embryonic as well as adult stem or progenitor cells [9]. All hedgehog proteins consist of a signal peptide, with a well-conserved N-terminal region serving the role of a signaling peptide. Hedgehog signal transduction is mediated by Smoothened (Smo), a G protein-coupled receptor with specificity toward Gi proteins [10]. Hedgehog-targeted cells present two components of the hedgehog-receptor complex on the cell surface: Smoothened (Smo) and Patched (Ptc). Ptc represses the activity of Smo, which regulates proteolytic processing of downstream zinc-finger transcription factors Gli1, 2, and 3. Although Gli2 rather seems to function as an activator, Gli3 acts mainly as a repressor [11].

We therefore hypothesized that MSCs overexpressing BMP-2 and/or IHH represent a powerful tool with a superior regenerative potential to native MSC. BMP transduction was chosen, as *de novo* bone formation *in vivo* is partly attributed to members of the transforming growth factor-β (TGF-β) superfamily, specifically the bone morphogenetic proteins (BMPs) [12] that were shown to have clinical utility in the treatment of recalcitrant nonunions [13] or spinal fusion [14] when delivered as recombinant proteins. The hedgehog pathway conversely plays a critical role in skeletogenesis that implicates mesenchymal precursors [15,16].

## Materials and methods

### MSC isolation and culture

Human MSCs were isolated as described previously [17]. After informed consent, freshly reamed trabecular bone from the acetabulum of patients undergoing total hip arthroplasty was transferred to 50 ml conical tubes (Greiner Bio-One, Frickenhausen, Germany) containing Dulbecco’s Modified Eagle Medium (DMEM; PAA Laboratories, Linz, Austria) supplemented with 10% fetal bovine serum (FBS; PAA) and antibiotics (50 IU penicillin/ml and 50 μg streptomycin/ml, PAA). The tubes were vortexed to disperse the marrow cells from the bone plugs. After centrifugation to high density pellets the released cells could be collected. The extracted cells were counted with a haemocytometer and plated at a density of 3 × 10^6^ cells per 175 cm^2^ tissue culture flask (Greiner Bio-One, Frickenhausen, Germany). After 2 days of culture, non-adherent cells were removed and the attached cells were washed twice with phosphate buffered saline (PBS). Cells were subsequently plated at a density of 3000/cm^2^ and expanded to the second or third passage for further experiments.

The study was approved by the institutional review board of the University of Würzburg in Germany.

### Generation and propagation of recombinant adenoviral vectors

First-generation, E1-, E3-deleted, serotype 5 adenoviral vectors carrying the cDNAs for human *IHH*, *BMP-2*, or green fluorescent protein (*GFP*) were constructed by using cre-lox recombination, as described previously [18,19]. The resulting vectors were designated Ad.IHH, Ad.BMP-2, and Ad.GFP. The vectors were propagated by amplification in 293/Cre8 cells and purified over cesium chloride gradients. After dialysis against 10 m*M* Tris-hydrochloric acid, pH 7.8, 150 m*M* sodium chloride, 10 m*M* magnesium chloride, and 4% sucrose, viral titers were estimated to be between 10^10^ and 10^11^ infectious viral particles (ip)/ml, as assessed by optical density measurements and a standard plaque assay.

### Transduction of MSC cultures and transgene expression

At confluence, MSC cultures were infected in 4 ml/175 cm^2^ of serum-free DMEM for 3 hours with 50 ip/cell of Ad.IHH, Ad.BMP-2, or in combination. Control cultures were similarly infected with similar doses of Ad.GFP or remained uninfected. After viral infection, the supernatant was aspirated and replaced with 20 ml of complete DMEM.

Media conditioned over a period of 24 hours were collected at several time points and assayed for IHH (Cusabio Biotech, Newark, DE, USA) and BMP-2 (R&D Systems) by using commercially available ELISA kits.

### Cell-proliferation assay

Adherent passage 3 MSCs were seeded in triplicate at 3,000 per square centimeter in flat-bottomed 24-well plates and maintained in 1-ml standard culture medium, consisting of DMEM supplemented with 10% FBS for 1, 3, 5, or 7 days in a humidified atmosphere (37°C, 5% CO_2_). At each time point, cells were washed twice with PBS and stored at −80°C until analysis. For analysis, samples were digested overnight with 0.5 mg/ml proteinase K in 1 × Tris-EDTA buffer at 55°C. DNA content for 100 μl of each sample in triplicate was measured and quantified by using a Qubit dsDNA BR assay kit, according to the protocol supplied by the manufacturer (Invitrogen). Fluorescence was measured at an excitation wavelength of 485 nm and an emission wavelength of 530 nm.

### Cell metabolic activity

Each cell type was seeded at a density of 3 × 10^3^ cells/cm^2^ in 100 μl culture media (phenol red free) in triplicate onto black 96-well plates and cultured for 4, 24, 48, 72, and 96 hours without media change. Two hours before the assay, 10 μl of AlamarBlue reagent (DAL1025; Biosource, Camarillo, CA, USA) were added to the culture medium at a final concentration of 10% (vol/vol). AlamarBlue added to medium served only as a negative control. Fluorescent signals (excitation 540 nm, emission 600 nm) were detected by using a fluorescence plate reader.

### Osteogenic induction of MSC cultures

Human MSCs were seeded at a density of 3,000 cells/cm^2^ into six-well polystyrene tissue-culture plates. After reaching confluence, mineralization was induced by supplementing the culture media with 50 μg/ml ascorbate-2-phosphate (Sigma-Aldrich), 10 m*M* β-glycerophosphate (Sigma-Aldrich), and 0.1 μ*M* dexamethasone (Sigma-Aldrich) over a period of 28 days. Controls were cultured in normal culture medium. Media were changed every 3 days.

### Alkaline phosphatase activity

Cultures were washed and incubated with 1 ml phenol red- and serum-free DMEM for 24 hours. Then 100 μl media supernatant was transferred in triplicate to a 96-well plate (Nunc). After 3 hours of incubation with 100 μl *p*-nitrophenylphosphate/0.2 *M* Tris buffer at a concentration of 1 mg/ml (Sigma), optical density at 405 nm was measured. ALP activity was normalized against the sample DNA content, determined by using a Qubit dsDNA BR assay kit (Invitrogen).

### Alizarin red S

Cultures were washed twice with double-distilled water (ddH_2_O), fixed with ice-cold methanol for 10 minutes, and incubated with 1% alizarin red S (Sigma-Aldrich) in ddH_2_O, pH 4.1, for 10 minutes at room temperature. After removal of the unincorporated dye, samples were washed at least 3 times with ddH_2_O and air-dried. Images of stained monolayers were captured with inverted-phase microscopy. The staining was quantified as described previously [20].

### Calcium assay

After 28 days, 2D cultures were washed with ddH_2_O and incubated with 800 μl of 10% acetic acid for 30 minutes at RT. Samples were heated to 85°C for 10 minutes, followed by a 10-minute incubation step on ice. Then 200 μl of 10% ammonium hydroxide was added to 500 μl of each sample. Triplicate 10-μl aliquots were transferred into a 96-well plate, to which 100 μl of a mono-ethanolamine buffer, pH 11.0, was added for a 3-minute incubation at 37°C, followed by 100 μl of *o*-cresolphthalein complexion (Calcium O-Cresolphthalein Kit; MTI Diagnostics, Wiesbaden, Germany) and incubation for 5 minutes at 37°C. To generate a standard curve, a standard dilution series in 10% acetic acid was used. Measurements were taken at λ = 570 nm.

### RNA isolation, primer design, and qRT-PCR

Total RNA was harvested in triplicate from control as well as differentiated samples at days 0, 7, 14, 21, and 28. Samples were isolated by using the NucleoSpin RNA II Kit (Machery-Nagel) according to the manufacturer’s instructions. cDNA was synthesized from 1 μg of total RNA by using the SuperScript III kit (Invitrogen). Quantitative RT-PCR for *alkaline phosphatase, osteopontin (OP), osteocalcin (OC), and runx2* was performed as described previously (Table 1) [20].

**Table 1:** qRT-PCR primer sequences

**Gene**	**Sequence (5′-3′; F, forward;, R, reverse)**	**Product (bp)**
*ALP*	F – GTACGAGCTGAACAGGAACAACG	151
	R – CTTGGCTTTTCCTTCATGGTG	
*OP*	F – TATGATGGCCGAGGTGATAG	133
	R – CATTCAACTCCTCGCTTTCC	
*OC*	F – TGACCACATCGGCTTTCAG	126
	R – AAGGGGAAGAGGAAAGAAGG	
*Runx2*	F – CTTCACAAATCCTCCCCAAG	147
	R – ATGCGCCCTAAATCACTGAG	
*RPS27A*	F – TCGTGGTGGTGCTAAGAAAA	141
	R – TCTCGACGAAGGCGACTAAT	

### Statistical analysis

Statistical analysis was performed by using ANOVA (IBM SPSS 19.0), and p values < 0.05 were considered significant.

## Results

**Figure 1:**
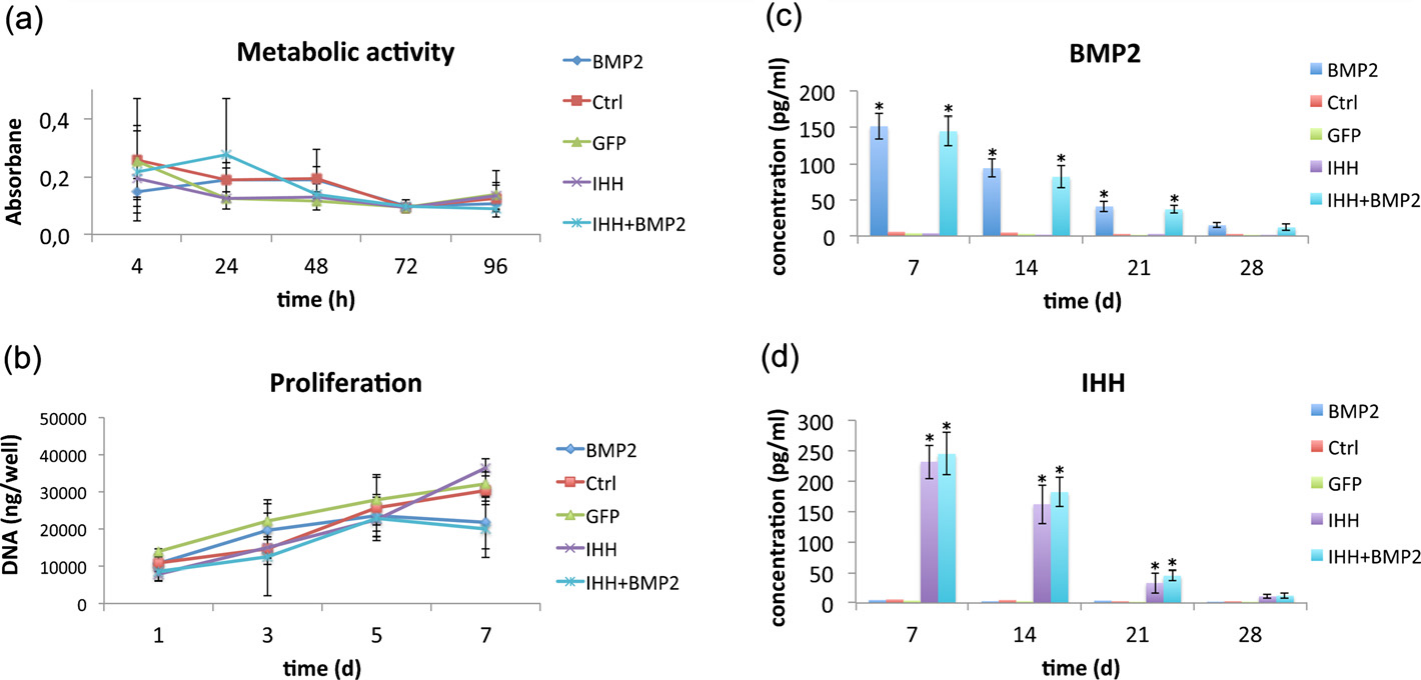
**Metabolic activity, proliferation, and levels of transgene products.** The figure illustrates the cell metabolic activity **(a)** and proliferation behavior **(b)** of native mesenchymal progenitor cells (Ctrl) and after adenoviral transduction with GFP, BMP-2, IHH, and BMP-2 with IHH, as determined with AlamarBlue and Quibit assays. Furthermore, the levels of BMP-2 **(c)** and IHH transgene products **(d),** determined with ELISA, are demonstrated. The asterisks indicate statistically significant differences compared with control cultures.

Viral transduction of primary human MSCs with Ad.IHH, Ad.BMP-2, Ad.GFP or Ad.IHH, and Ad.BMP-2 in combination led to a slight but nonsignificant decrease in metabolic activity over the first 72 hours after transduction (Figure 1a). Genetic modification by viral transduction did not negatively affect cell proliferation. All transduced cells proliferated well when compared with native nonmodified primary MSCs, and no significant differences were observed between groups. Nevertheless, a tendency toward an increased proliferative capacity was observed after BMP-2 or IHH transduction (Figure 1b).

Monolayer cultures infected with Ad.IHH, Ad.BMP-2, Ad.GFP or Ad.IHH, and Ad.BMP-2 in combination at 50 ip/cell generated high levels of transgene products at day 7 of culture, with doses ranging from 130 to 160 pg/ml for BMP-2 and 160 to 250 pg/ml for IHH. The amount of transgene declined over time, as evidenced by ELISA measurements (Figure 1c and d).

**Figure 2:**
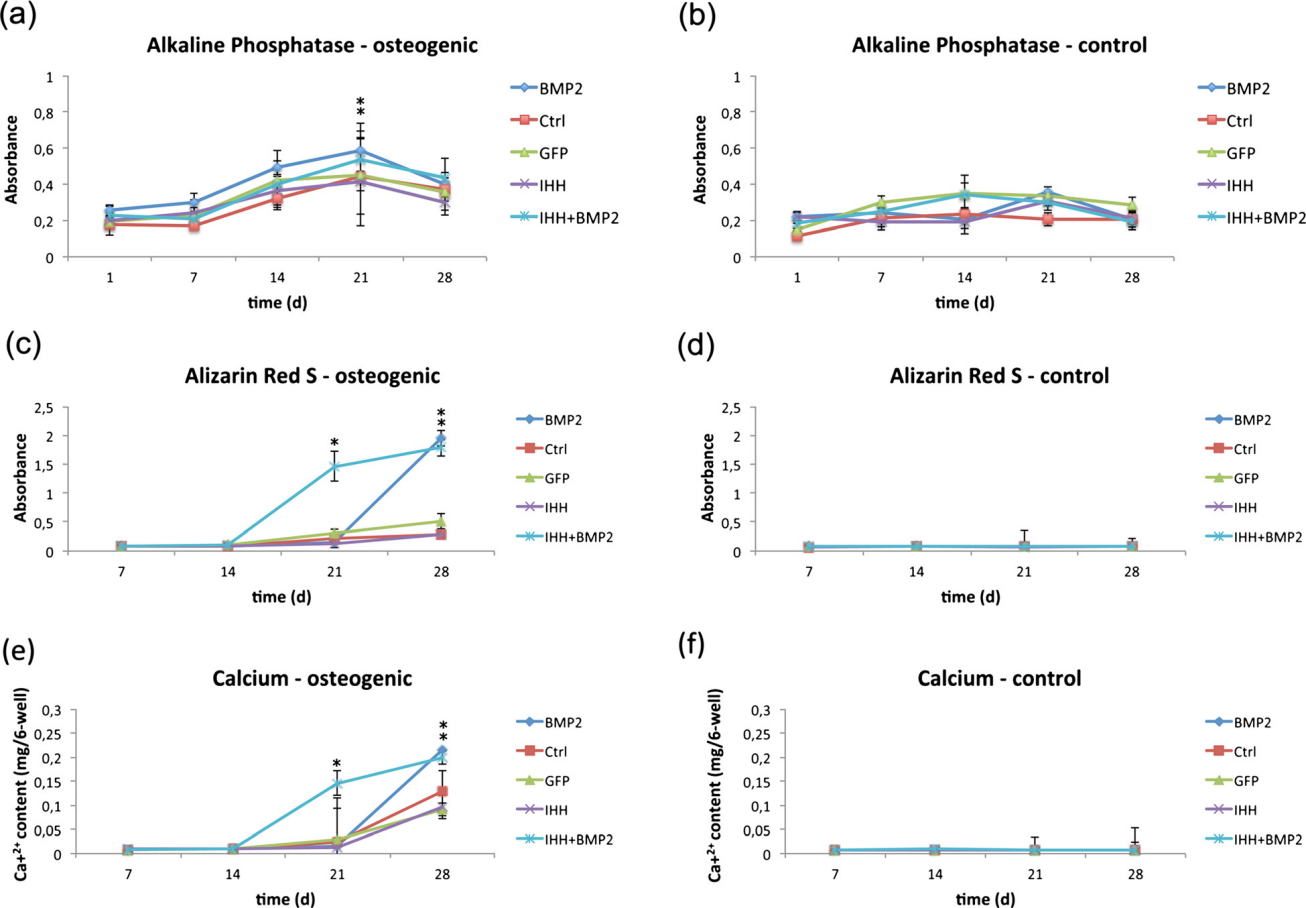
**ALP activity and mineralization.** The figure shows the alkaline phosphatase activity of osteogenic **(a)** and control **(b)** monolayer cultures of native (Ctrl) and transduced mesenchymal progenitor cells. Moreover, the extracellular matrix mineralization of osteogenically induced **(c, e)** and control cultures **(d, f)** over time is depicted. Asterisks indicate statistical significance.

Induction with osteogenic media caused an increase of alkaline phosphatase (ALP) activity in all groups when compared with control cultures. The level of enzyme activity displayed a typical increase-and-decrease pattern. ALP activity appeared to be significantly higher on day 21 in BMP-transduced cells and cells transduced with BMP-2 and IHH in combination, when compared with native MSCs (Figure 2a and b). A statistically significant difference between the BMP and BMP + IHH groups, however, could not be determined.

**Figure 3:**
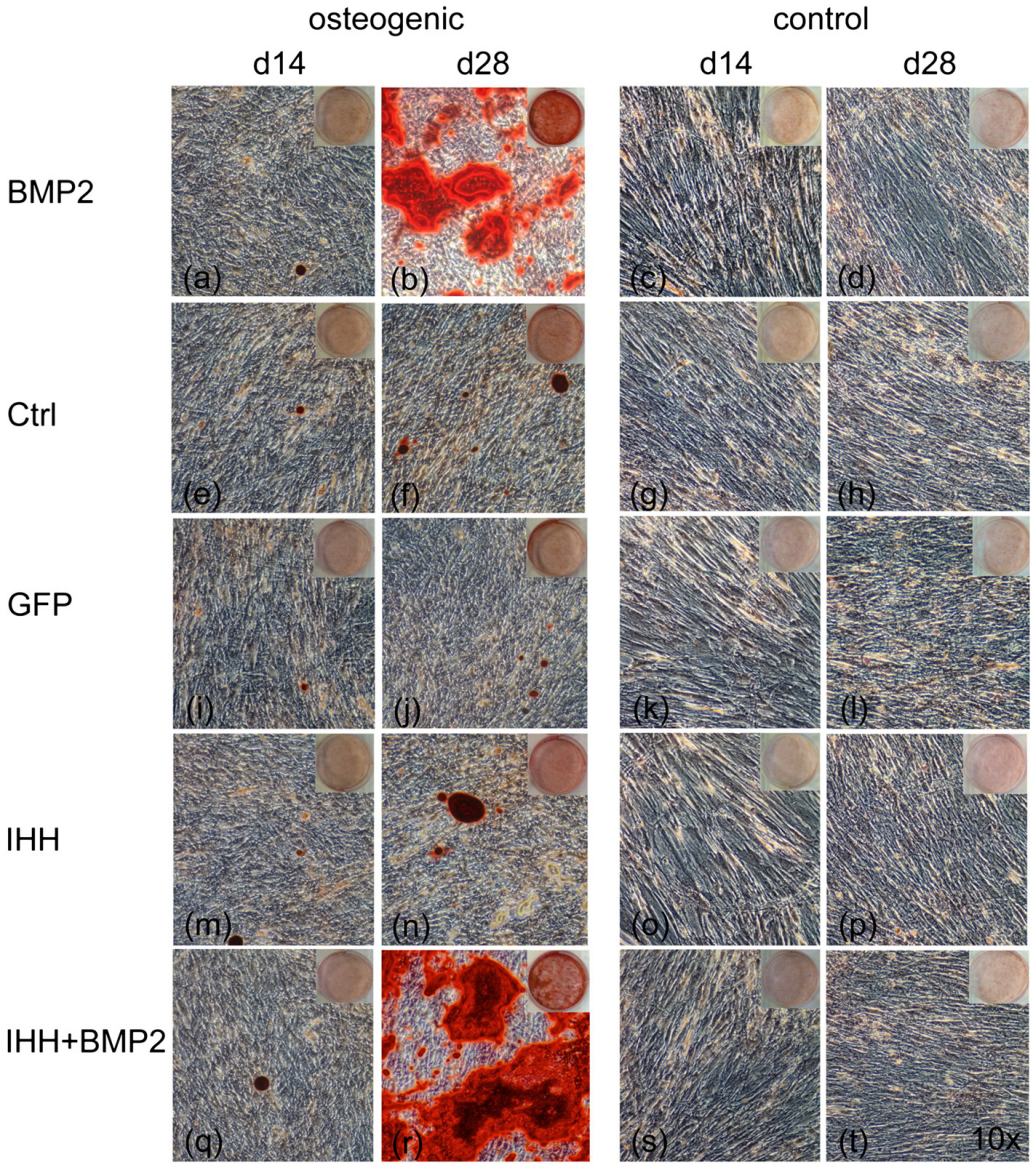
**Monolayer mineralization.** Alizarin red S stainings on days 14 and 21 of monolayer cultures transduced with GFP, BMP-2, IHH, and BMP-2 with IHH maintained with osteogenic (left panel) and control (right panel) media. Nonmodified mesenchymal progenitor cells (Ctrl) served as controls. Magnification, ×10.

Compared with controls, osteogenic induction furthermore increased the deposition of mineralized extracellular matrix, which started around day 14 of culture. Mineralization was accelerated in monolayers transduced with BMP-2 and IHH in combination, when compared with all other groups, to result in a significantly higher mineralization on day 21. Similar levels of mineralization were observed in osteogenic cultures of BMP- and BMP + IHH- overexpressing cells on day 28. These levels of mineralized matrix deposition were measured to be significantly higher than those in the other experimental groups (Figure 2c and d, Figure 3). The quantification of the incorporated alizarin red dye correlated very well with the results of the calcium assay, measuring calcium amounts ranging from 0.025 to 0.21 mg/six wells (Figure 2e and f).

**Figure 4:**
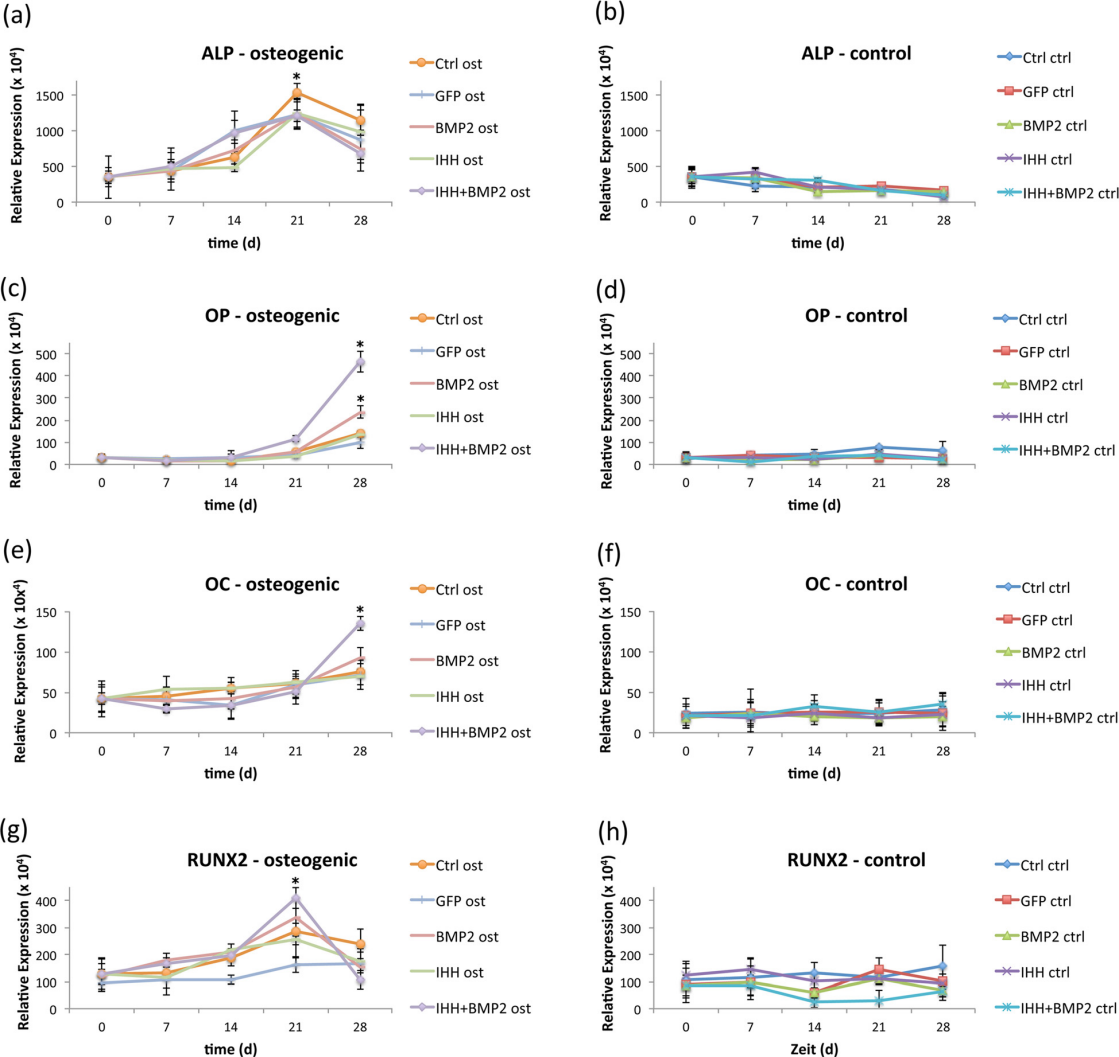
**RT-PCR analysis.** Outcomes of the qRT-PCR measurements for the osteogenic marker genes *alkaline phosphatase* (*ALP***(a)**), *osteopontin* (*OP*, **(b)**), *osteocalcin* (*OC*, **(c)**), and *runx2***(d)** in osteogenic (left panel) and control cultures (right panels).

ALP activity measured on days 0, 7, 14, and 28 displayed a typical increase/decrease pattern significantly increasing toward day 21 in osteogenically induced MSCs (*P <* 0.05) when compared with initial levels on day 0. On the day of maximum *ALP* expression (day 21), no significant differences were observed between the osteogenic groups. *ALP* expression in the respective noninduced controls remained on basal levels (Figure 4a and b).

*OP* and *OC* both showed a significant increase in expression over time for all groups cultured in osteogenic media compared with day 0 and cultures in control media. On day 28, a significantly higher expression was observed for both genes in the groups transduced with BMP-2 and BMP-2 + IHH, whereas the highest expression was determined for cells simultaneously overexpressing BMP-2 and IHH (Figure 4c through f).

As with *ALP, runx2* showed a significantly increasing expression pattern up to day 21 in osteogenic cultures to decrease further toward day 28. The expression of the transcription factor on day 21 was significantly higher in the BMP-2 and BMP-2 + IHH groups. In noninduced controls, runx2 expression remained on basal levels (Figure 4g and h).

## Discussion

Sufficient bone healing is strongly dependent on the efficient and coordinated migration, proliferation, and differentiation of mesenchymal progenitor cells at the site of injury. Well-orchestrated molecular pathways precisely control this process. Hedgehog signaling is known to contribute to regulate tissue self-renewal processes, and the migration, differentiation, and cell-fate commitment of embryonic as well as adult stem or progenitor cells [16].

After binding their cell-surface receptors patched-1 and smoothened, hedgehog signal transduction occurs via members of the Gli family of zinc-finger transcription factors (Gli1, 2, and 3) [21]. The hedgehog pathway was previously reported to be involved in skeletal morphogenesis [15]. It could be demonstrated that IHH-null mouse embryos lack the typical perichondral expression of osteogenic markers such as *runx2, collagen type I, and ALP*[22]. It was therefore suggested that IHH signaling influences early osteoblastogenesis by modulation of *runx2*.

In the present study, we investigated whether adenoviral delivery of Indian hedgehog (IHH) is suitable to increase the osteogenic potential adult human mesenchymal progenitor cells. Our results indicate that with our specific experimental settings, IHH alone fails to do so, as (when compared with controls), no increased mineralization was observed in osteogenically induced MSC cultures transduced with IHH. However, when combined with simultaneous adenoviral BMP-2 transduction, IHH appeared to accelerate mineralization and to promote the expression of the osteogenic markers *osteopontin and osteocalcin*. Both mineralization and marker expression were greater than the sum of effects observed in cultures transduced solely with BMP-2 or IHH. Consequently, IHH and BMP-2 seemed to act synergistically.

Bone morphogenetic proteins are well-studied members of the TGF-β superfamily. Among several of their functions, BMPs induce the formation of both bone and cartilage by stimulating the cellular events of mesenchymal progenitor cells [23]. However, only a subset of BMPs, specifically BMP-2, -4, -7, and -9, have osteoinductive activity [8].

As mentioned earlier, in conjunction with BMP-2, IHH appears to have a synergistic effect on osteogenesis in the mesenchymal cell type used in our study. Conversely, sonic hedgehog (SHH)-induced osteoblastic differentiation seems to require functional BMP signaling [24]. Consequently, hedgehog (HH) proteins and BMP-2 were shown to induce ALP activity synergistically in KS483 cells [25]. Moreover, both IHH and SHH treatment were shown to stimulate ALP activity synergistically with BMP-2 in C3H10T1/2 cells and MC3T3-E1 cells [26]. Similarly, recombinant N-terminal SHH could cause a greater number of cells to respond to BMP-2, increasing ALP activity, as demonstrated in C3H10T1/2, ST2, and primary mouse calvaria cells [24]. ALP is considered a marker for osteoblastic activity *in vitro*. ALP activity usually displays a typical increase/decrease pattern [27], which is in line with our findings.

It was also reported that hedgehog influence might depend on the extent of terminal cell differentiation. Rather, committed osteogenic lineages, such as preosteoblastic MC3T3-E1 cells and osteoblastic cell lines, did not respond to SHH in the presence of BMP-2. C3H10T1/2 cells that display pluripotent characteristics, conversely did show a response [24]. Consequently, the contribution of HH to osteoblastogenesis appears of greater importance in the early stages of differentiation. This may have contributed to the positive effects observed in our study, in which undifferentiated, multipotent MSCs were used. Study reports describing mouse perichondrial osteoblastic progenitors to undergo chondrogenesis rather than osteoblastogenesis in absence of IHH signaling [28] further support this hypothesis.

Notably, all HH proteins consist of a signal peptide with a highly conserved N-terminal region. As a result, HH ligands are functionally interchangeable [21], and observations made for SHH can most likely be transferred to IHH.

The literature suggests that the effect of HH on osteoblast differentiation, with or without BMP, may differ between species. HH, in synergy with BMP, appears to promote osteoblastogenesis in mice, nonsynergistic or even antagonistic effects may occur in other species. In a rat model, SHH enhanced endochondral ossification mediated by *runx2*, but no increase in ossification was observed when low amounts of BMP-4 were simultaneously administered [29]. In human adipose-derived stem cells, SHH even inhibited osteogenesis, as measured by decreased *ALP and runx2* expression [30].

*Runx2*, also referred to as core-binding factor subunit alpha-1 (*CBF-α-1*), is considered a master gene for osteoblast differentiation, matrix production, and mineralization during bone formation. In our study, *runx2* was significantly upregulated through the synergistic effects of BMP-2 and IHH transduction. *Runx2* expression is necessary to achieve differentiation and activation of osteoblasts. Its null mutation in mice exhibits the complete absence of bone [31]. *Runx2* expression is upregulated in proliferative chondrocytes, and it is expressed early in osteoblastic differentiation. Multiple signal-transduction pathways can stimulate *runx2* gene expression [32]. *Runx2*, conversely, can directly control the expression of essential bone-associated extracellular matrix protein genes mediated by a direct binding site (osteoblast-specific cis-acting element (*OSE2*)). *OSE2* can be found in the promoter region of various osteoblast-specific genes, such as *OC, OP, BSP*, and *collagen type I*[33,34].

The noncollagenous extracellular matrix components, such as OC and OP, contribute to provide bone with its physical and chemical properties [35]. OC is partly incorporated into the bone matrix, partly delivered to the circulatory system, and considered a late and specific osteogenic marker determining terminal osteoblast differentiation regulating bone crystal formation [36]. We observed a significantly higher *OC* expression on day 28 after the combined adenoviral transduction of BMP-2 and IHH.

OP is yet another key modulator of bone function. In our cell-culture experiments, the simultaneous action of BMP-2 and IHH resulted in a significant upregulation of *OP* expression. OP is considered an early osteogenic marker with affinity for calcium and binding sites for integrin receptors through a RGD motif. OP, therefore, functions as an important protein for matrix-cell interaction [37]. Through the RGD motif, osteopontin mediates the attachment and activation of osteoclasts [38] and can facilitate attachment of bone cells to mineralized tissue surfaces [39].

## Conclusion

Our results generally suggest that adenoviral modification of bone growth-associated cells by simultaneous transduction with BMP-2 and IHH represents a promising way to stimulate osseous healing. Nevertheless, these results must be confirmed by thorough *in vivo* investigations.

Furthermore, viral transduction is eyed skeptically in treating nonlethal conditions in humans [40].

Adenoviruses are DNA viruses. Their genomes rarely integrate into host-cell DNA. Nevertheless, transgene expression by these viruses is congruent with the requirements of healing bone. With adenoviruses, the chosen recombinant vector can be produced at high titer. Furthermore, adenovirus displays a high infectivity toward many cell types [41].

Disadvantages of adenovirus certainly include its high antigenicity [41], as many individuals possess circulating, neutralizing antibodies. These antibodies might reduce the effectiveness of adenovirus-based gene therapy. Moreover, the caused inflammatory response may negatively influence the healing process [42]. No consensus exists concerning the most appropriate vectors and transgenes, and, consequently, further research is needed.

## Abbreviations

2D: Two-dimensional; ABG: Autologous bone graft; ALP: Alkaline phosphatase; BMP-2: Bone morphogenetic protein 2; BSP: Bone sialo protein; cDNA: Complementary DNA; ddH2O: Double-distilled water; DMEM: Dulbecco modified eagle medium; DNA: Deoxyribonucleic acid; EDTA: Ethylenediaminetetraacetic acid; FBS: Fetal bovine serum; GFP: Green fluorescent protein; HH: Hedgehog; IHH: Indian hedgehog; ip: Infectious viral particle; MSC: Mesenchymal stem cell; OC: Osteocalcin; OP: Osteopontin; OSE2: Osteoblast-specific cis-acting element; PBS: Phosphate-buffered saline; RGD: Arginine-glycine-aspartic acid; RNA: Ribonucleic acid; SHH: Sonic hedgehog.

## Competing interests

The authors declare that they have no competing interests.

## Authors’ contributions

JS, FG, and PP carried out the molecular studies. JCR and VMCQ carried out the immunoassays. JCR, PP, and AFS participated in the design of the study and performed the statistical analysis. JCR, MR, and UN conceived of the study and participated in its design and coordination. JCR and VMCQ drafted the manuscript. All authors read and approved the final manuscript.
